# Efficacy and Safety of Curcumin Supplement on Improvement of Insulin Resistance in People with Type 2 Diabetes Mellitus: A Systematic Review and Meta-Analysis of Randomized Controlled Trials

**DOI:** 10.1155/2021/4471944

**Published:** 2021-08-24

**Authors:** Tianqing Zhang, Qi He, Yao Liu, Zhenrong Chen, Hengjing Hu

**Affiliations:** ^1^The First Affiliated Hospital, Department of Cardiovascular Medicine, Hengyang Medical School, University of South China, Hengyang, Hunan Province, China; ^2^People's Hospital of Ningxiang City, Ningxiang City, Hunan Province, China; ^3^Institute of Cardiovascular Disease and Key Lab for Arteriosclerology of Hunan Province, University of South China, Hengyang, Hunan, China

## Abstract

**Background:**

Diabetes is a major public health concern. In addition, there is some evidence to support curcumin as part of a diabetes treatment program.

**Methods:**

Data from randomized controlled trials were obtained to assess the effects of curcumin versus placebo or western medicine in patients with type 2 diabetes mellitus (T2DM). The study's registration number is CRD42018089528. The primary outcomes included homeostasis model assessment-insulin resistance (HOMA-IR), glycosylated hemoglobin (HbAlc), total cholesterol (TC), and triglyceride (TG).

**Results:**

Four trials involving 453 patients were included. The HOMA-IR of curcumin group is lower in Asia (WMD: −2.41, 95% CI: −4.44 to −0.39, *P*=0.02) and the Middle East subgroups (WMD: −0.60, 95% CI: −0.74 to −0.46, *P* < 0.00001). The HbAlc in the curcumin group is lower than that in the control group (WMD: −0.69; 95% CI: −0.91, −0.48; *P* < 0.0001). The TC and TG levels of the curcumin group are lower in the Asia subgroup (TC: WMD: −23.45, 95% CI: −40.04 to −6.84, *P*=0.006; TG: WMD: −54.14, 95% CI: −95.71 to −12.57, *P*=0.01), while in the Middle East the difference was of not statistically significant (TC: WMD: 22.91, 95% CI: −16.94 to 62.75, *P*=0.26; TG: WMD: −4.56, 95% CI: −19.28 to 10.16, *P*=0.54).

**Conclusion:**

Based on the current evidence, curcumin may assist in improving the insulin resistance, glycemic control, and decreased TG and TC in patients with T2DM.

## 1. Introduction

As a serious metabolic disease, diabetes affects about 5% of the world's people. Epidemiological data show that the number of people with diabetes is expected to increase dramatically to 592 million by 2035 [1]. 12% of global health expenditure is spent annually on diabetes and its complications [2]. Diabetes is divided into different types, wherein type 1 and type 2 diabetes accounted for more than 90% of all cases. Among these types of diabetes, type 2 diabetes mellitus (T2DM) causes metabolic abnormalities and serious complications that have a profound impact on the patient's lifespan and quality of life. T2DM is mainly characterized by insulin resistance and hyperglycemia [3]. Its common complications include microvascular disease (diabetic retinopathy, diabetic nephropathy, and diabetic neuropathy), macrovascular disease (diabetic heart disease, diabetic cerebrovascular disease, and peripheral vascular disease) [4, 5], and increased risk of cancer [6, 7]. At present, T2DM has a variety of therapeutic drugs, such as human insulin preparation, alpha glucosidase inhibitor, dipeptidyl peptidase-4 inhibitor, incretin analogue, biguanide, insulin secretagogue, insulin sensitizer, and intestinal lipase inhibitor [8, 9]. However, the currently used therapies have many side effects such as hypoglycemia, gastrointestinal problems, and weight gain [8]. Therefore, new drugs and natural compounds are constantly being tested to prevent and treat diabetes better [10].

Curcumin is a chemical component extracted from the rhizome of some plants. It has a series of effects such as blood lipid lowering, antitumor, anti-inflammatory, and antioxidation [11, 12] and has been used as a food flavoring agent, preservative, and ancillary medication for some diseases (such as heart disease and tumors) [13, 14]. In the treatment of diabetes, there is also evidence to support curcumin as a part of the diabetes treatment program [14, 15]. At present, many randomized controlled trials (RCTs) on the treatment of T2DM with curcumin have been published [11, 16–22], but there is still no systematic review and meta-analysis to assess the effects and safety of curcumin. Therefore, we decide to perform a systematic review and meta-analysis for the first time to evaluate the clinical effects of curcumin on T2DM.

## 2. Materials and Methods

### 2.1. Protocol

Study selection, assessment of eligibility criteria, data extraction, and statistical analyses were performed based on a predefined protocol registered on PROSPERO CRD42018089528 (see supplementary materials) [23].

### 2.2. Search Strategy and Selection Criteria

We searched the English database and the Chinese database from the beginning of their establishment to September 3, 2020. The English database includes EMBASE, Medline Complete, the Cochrane Library (until Issue 9, 2020), ClinicalTrials, PubMed, and Web of Science. The Chinese database includes the Chinese Science and Technology Periodical Database (VIP), Chinese Biomedical Database (CBM), Wan Fang Database (Chinese Ministry of Science and Technology), and China National Knowledge Infrastructure Databases (CNKI). The search strategy for PubMed is presented in Table 1 as an example.

Studies meeting the inclusion criteria would be included in this review: (1) participants: patients with type 2 diabetes mellitus; (2) intervention: curcumin with no limits on the type, dose, frequency, and so on; (3) comparisons: Western medicine, blanks, or placebo; (4) outcomes: primary outcomes: homeostasis model assessment-insulin resistance (HOMA-IR), glycosylated hemoglobin (HbAlc), total cholesterol (TC), and triglyceride (TG); secondary outcomes: body mass index (BMI), low-density lipoprotein cholesterol (LDL-C), high-density lipoprotein cholesterol (HDL-C), fasting glucose, and fasting insulin; (5) study type: randomized controlled trials (RCTs) with no limits on the manner by which randomization has been achieved on blinding or on the language of publication. Studies meeting the exclusion criteria would be excluded: (1) not T2DM patients; (2) the participant is not human; (3) nonoriginal research literature; and (4) non-RCT.

### 2.3. Data Extraction

Two reviewers independently extracted the data and it was checked by a third reviewer. When there is a disagreement, a consensus is reached through mutual discussion and negotiation with all reviewers. The extracted data include basic information (author, publication time, age of the research object, etc.), sample size, intervention measures, dose, intervention time, outcomes, etc. [24].

### 2.4. Study Quality Assessment

The risk of bias of RCTs was assessed by using the risk of bias assessment tool based on the Cochrane Handbook [25]. Two reviewers independently assessed the risk of bias. When there is a disagreement, a consensus is reached through mutual discussion and negotiation with all reviewers. The risk of bias is divided into three levels: high risk, low risk, and unclear. The content of the evaluation includes random sequence generation, allocation concealment, blinding, incomplete outcomes, selective reporting, and other bias.

### 2.5. Statistical Analysis

The data were analyzed by RevMan 5.3 software. Cochrane's *Q* and *I*^2^ test were used to judge the heterogeneity of different studies. If there is good homology between studies (*I*^2^ < 50%, *P* > 0.1), the fixed-effects model is used for meta-analysis. If there is heterogeneity between studies (*I*2 > 50%, *P* < 0.1), we first find the source of heterogeneity, conduct subgroup analysis, and then choose random-effects model or give up meta-analysis [26]. The dichotomous variable measure was summarized by risk ratio (RR) with a 95% confidence interval (CI). The continuous outcomes underwent meta-analysis using weight mean differences (WMD) and 95% CI. If the units of outcomes are different, or the value difference between RCTs is more than 10 times, the standard mean differences (SMD) and 95% CI are used according to the situation.

### 2.6. Sensitivity Analysis and Publication Bias Detection

STATA 15.0 was utilized for sensitivity analysis and publication bias detection. Studies with RCTs ≥5 were evaluated for publication bias. The outcomes with *P* > 0.1 were thought to have publication bias. The outcomes that meet the following conditions are all subjected to sensitivity analysis: (1) random-effects model is used; (2) number of included RCTs ≥ 3; and (3) the results of the fixed-effects model are inconsistent with the results of the random-effects model (whether it is a subgroup result or a summary result).

## 3. Results

### 3.1. Results of the Search

**Figure 1:**
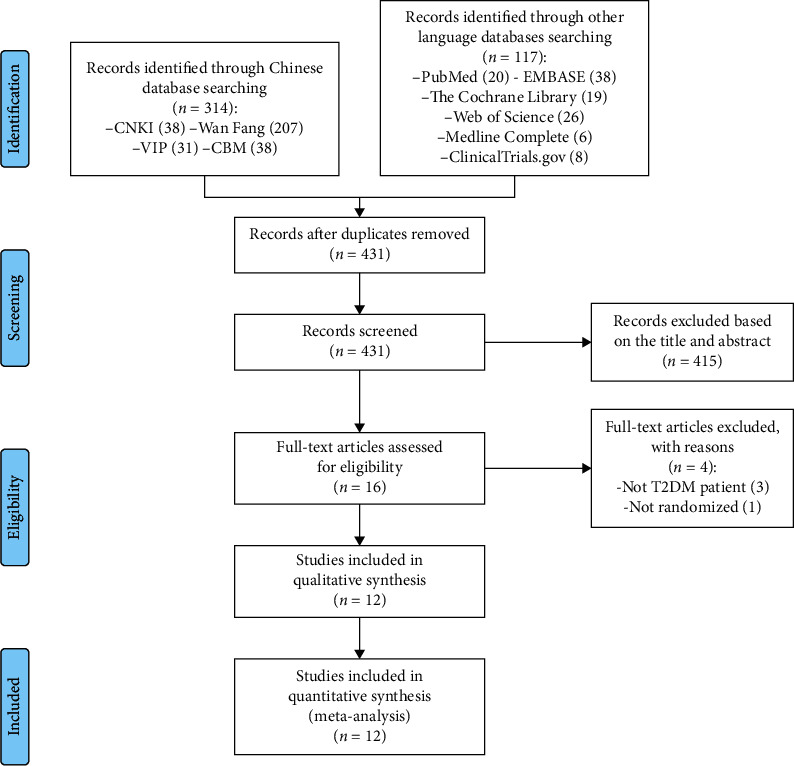
Flow diagram of searching and article selection.

The total records identified through database searching and other sources were 431. Sixteen records were included after initial identification. Four records were excluded: Yang et al. is not RCT [27]; and the participants in the remaining 3 records are not only T2DM patients [28–30]. Eventually, 12 records were included to undergo analysis (Figure 1).

### 3.2. Description of Included Trials

Twelve records met the inclusion criteria [11, 16–22, 31–34]. Of the 12 records, 4 records [16–19] are from one RCT: Panahi et al., 2 records [20, 21] are from the same RCT: Na et al, and 2 records [31, 32] are from one RCT: Asadi et al. Hence, a total of 7 RCTs are included in this review. All of them were parallel-group RCTs. Study characteristics are presented in Table 2.

### 3.3. Risk of Bias of Included Studies

**Figure 2:**
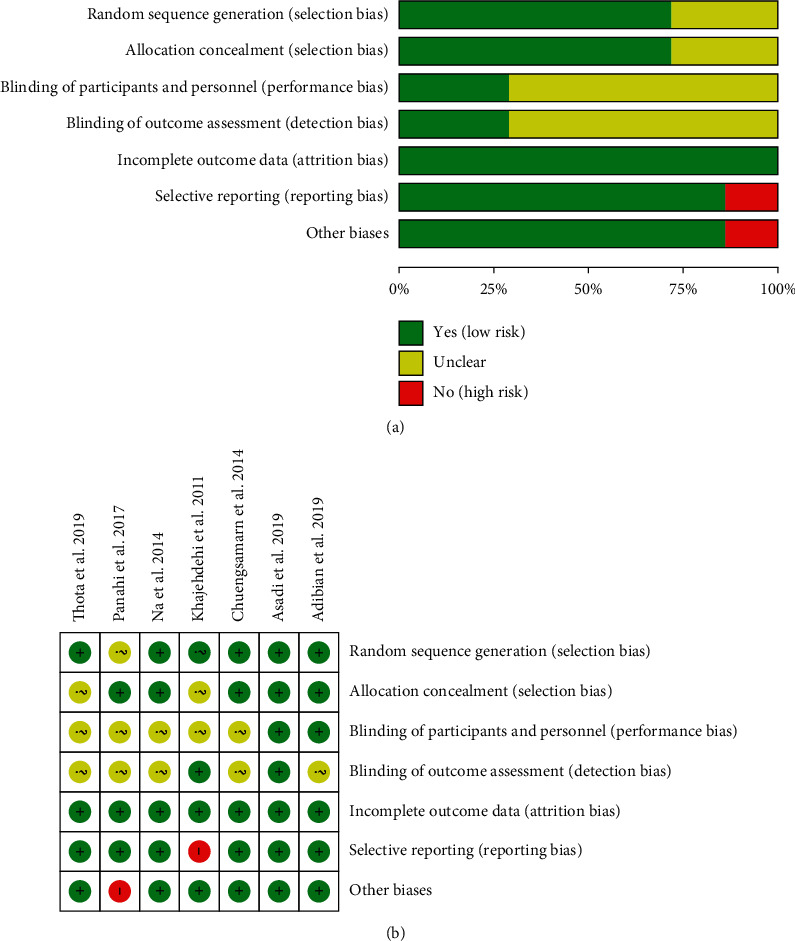
(a) Risk of bias graph. (b) Risk of bias summary.

The summary and graph of risk of bias are shown in Figure 2.

#### 3.3.1. Sequence Generation

Panahi et al. [16–19] and Khajehdehi et al. [22] failed to describe the method of randomization, and we therefore rated it as having an unclear risk of bias. Na et al. [20, 21], Chuengsamarn et al. [11], and Thota et al. [33] utilized a computer-generated random list, and Asadi et al. [31, 32] and Adibian et al. [34] utilized block randomization, so they were thought to have low risks of bias.

#### 3.3.2. Allocation Concealment

Khajehdehi et al. [22] and Thota et al. [33] did not describe an acceptable method of allocation concealment; therefore, they were rated as having an unclear risk of bias. Panahi et al. [16–19], Na et al. [20, 21], Asadi et al. [31, 32], and Adibian et al. [34] utilized the capsules in the same shape, size, and color to contain curcumin and placebo; Chuengsamarn et al. [11] used opaque and consecutively numbered envelopes. Hence, these five RCTs considered to have allocation concealment were rated as having low risks of bias.

#### 3.3.3. Blinding, Incomplete Outcome Data, and Selective Reporting

All RCTs claimed to use blinding, but only Asadi et al. [31, 32] described the implementation process for researchers and participants, so only its double blinding was rated as a low risk of bias. Four RCTs [11, 16–21, 33] did not describe the implementation process for both researchers and participants, and they were rated as high risk of bias. Khajehdehi et al. [22] described blinding to researchers, so its blinding of outcome assessment (detection bias) was rated as low risk of bias. Adibian et al. [34] described blinding of participants, so the blinding of participants and personnel (performance bias) was rated as low risk of bias.

#### 3.3.4. Incomplete Outcome Data and Selective Reporting

The incomplete outcome data of all RCTs are rated as low risk of bias because the number of missing persons and the reasons for the missing between groups is balanced. One RCT (Khajehdehi et al. [22]) failed to provide all outcomes mentioned in its protocols; thus, we thought its risk of bias was high. The others 6 RCTs reported study's prespecified outcomes that are of interest in the review; and their risks of bias were low.

#### 3.3.5. Other Potential Bias

Panahi et al. [16–19] claimed that one of the authors had a conflict of interest and was therefore assessed as a high risk of bias. Other sources of bias in the other 6 RCTs were not found; therefore, the risks of other bias of the RCTs were low.

### 3.4. Primary Outcomes

#### 3.4.1. Homeostasis Model Assessment-Insulin Resistance

**Figure 3:**
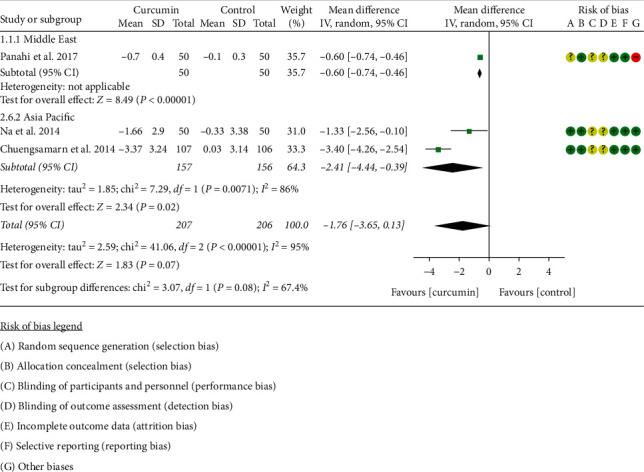
Homeostasis model assessment-insulin resistance.

Four RCTs [11, 16–21, 34] reported HOMA-IR. However, the data in Adibian et al. [34] cannot be extracted; hence, the data in four RCTs (207 participants in the curcumin group and 206 participants in the control group) were extracted. Due to the high heterogeneity, three RCTs were subdivided into two subgroups according to the region of the patients. After subdivision, the heterogeneity was still high in each subgroup (Asia Pacific: *I*^2^ = 86%, *P*=0.07; the Middle East: not applicable) among the RCTs. The random-effects model was used. The HOMA-IR in the curcumin group was lower than that in the control group in the Middle East subgroup (WMD: −0.60, 95% CI:−0.74 to −0.46, *P* < 0.00001) and the Asia subgroup (WMD: −2.41, 95% CI −4.44 to −0.39, *P*=0.02). However, the summary result showed that the difference in HOMA-IR between two groups was of no significance (WMD: −1.76, 95% CI:−3.65 to 0.13, *P*=0.07) (Figure 3).

#### 3.4.2. Glycosylated Hemoglobin

**Figure 4:**
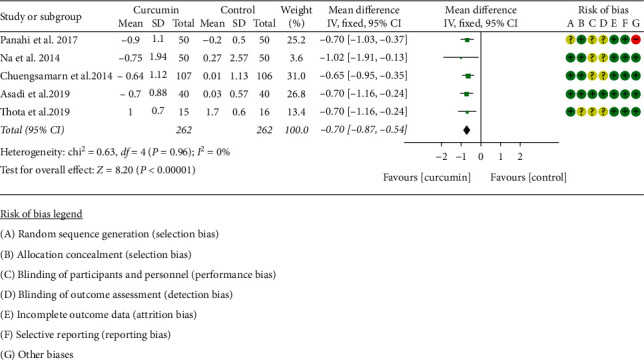
Glycosylated hemoglobin.

Six RCTs [11, 16–21, 31–34] reported HbAlc. However, the data in Adibian et al. [34] cannot be extracted; hence, the data in 5 RCTs including 262 participants in the curcumin group and 262 participants in the control group were extracted. Due to the high heterogeneity (*I*^2^ = 0%, *P*=0.96), the fixed-effect model was utilized. The results showed that, compared with the control group, curcumin can reduce HbAlc levels (WMD: −0.70; 95% CI: −0.87, −0.54; *P* < 0.0001) (Figure 4).

#### 3.4.3. Total Cholesterol

**Figure 5:**
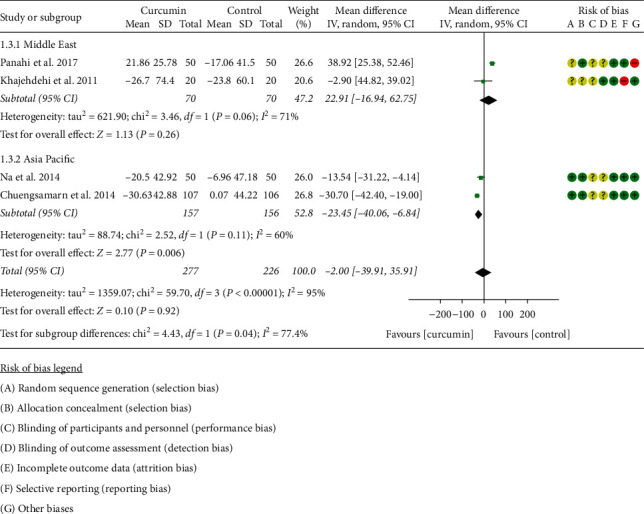
Total cholesterol.

Five RCTs [11, 16–22, 34] reported TC; however, Adibian et al. [34] used the same symbol for TC and TG, so it is not clear which group of data is TC, and the data cannot be extracted. The data in four RCTs containing 227 participants in the curcumin group and 226 participants in control group were extracted. Due to the high heterogeneity, four RCTs were subdivided into two subgroups according to the region of the patients. After subdivision, the heterogeneity was still high in each subgroup (Asia Pacific: *I*^2^ = 60%, *P*=0.11; the Middle East: *I*^2^ = 71%, *P*=0.06) among the RCTs. The random-effects model was used. The difference between two groups in the Asia subgroup has statistical significance (WMD: −23.45, 95% CI:−40.04 to −6.84, *P*=0.006), but it did not have statistical significance in the Middle East subgroup (WMD: 22.91, 95% CI: −16.94 to 62.75, *P*=0.26). The summary result also showed that the difference between two groups was of no significance (WMD: −2.00, 95% CI:−39.91 to 35.91, *P*=0.92) (Figure 5).

#### 3.4.4. Triglyceride

**Figure 6:**
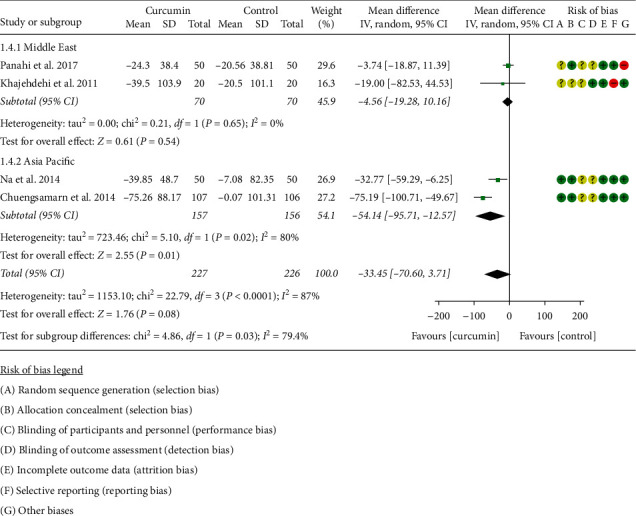
Triglyceride.

Five RCTs [11, 16–22] reported TG; however, Adibian et al. [34] used the same symbol for TC and TG, so it is not clear which group of data is TG, and the data cannot be extracted. The data in four RCTs involving 227 participants in the curcumin group and 226 participants in the control group were extracted. Due to the high heterogeneity, four RCTs were subdivided into two subgroups according to the region of the patients. After subdivision, the heterogeneity was still high in the Asia subgroup (*I*^2^ = 80%, *P*=0.02) but low in the Middle East subgroup (*I*^2^ = 60%, *P*=0.11) among the RCTs. The random-effects model was used in the Asia subgroup. The difference between two groups in the Asia subgroup has statistical significance (WMD: −54.14, 95% CI: −95.71 to −12.57, *P*=0.01), but it did not have statistical significance in the Middle East subgroup (WMD −4.56, 95% CI: −19.28 to 10.16, *P*=0.54). The summary result also showed that the difference between two groups was of no significance (WMD: −33.45, 95% CI: −70.60 to 3.71, *P*=0.08) (Figure 6).

### 3.5. Secondary Outcomes

#### 3.5.1. Body Mass Index

Three RCTs [11, 16–19, 31, 32] reported BMI. Due to the high heterogeneity (*I*^2^ = 89%, *P*=0.0002), the statistical analysis was abandoned according to the Cochrane Handbook for Systematic Reviews of Interventions. Panahi et al. [16–19] found that there was a statistically significant difference in BMI changes between the curcumin group and the control group (−0.49 ± 0.52 versus 0.24 ± 0.73; *P* < 0.001). Chuengsamarn et al. [11] also found that there was a statistically significant difference in BMI changes between the curcumin group and the control group (−1.97 ± 5.38 versus 0.16 ± 4.32; *P* < 0.001). However, Asadi et al. [31, 32] found that there were no significant differences in terms of BMI and weight between the two groups.

#### 3.5.2. Fasting Blood Glucose and Fasting Insulin

**Figure 7:**
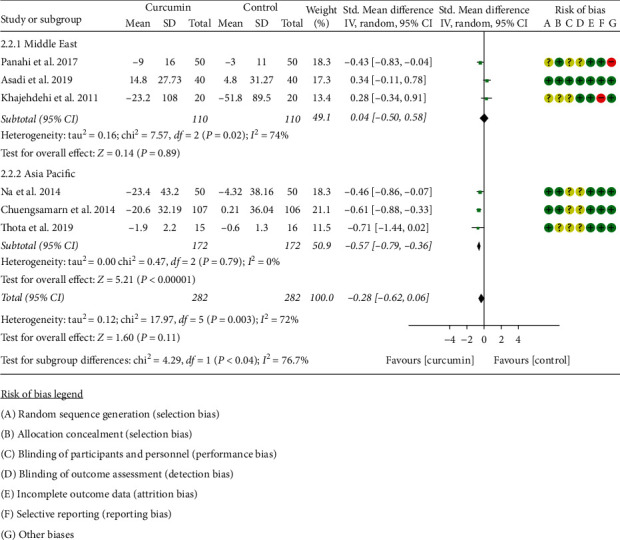
Fasting blood glucose.

All RCTs [11, 16–22, 31–34] reported fasting blood glucose. However, the data in Adibian et al. [34] cannot be extracted; hence, the data in 6 RCTs include 282 participants in the curcumin group and 282 participants in the control group. Due to the high heterogeneity, 6 RCTs were subdivided into two subgroups according to the region of the patients. After subdivision, the heterogeneity was low in the Asia Pacific subgroup but high in the Middle East subgroup (Asia: *I*^2^ = 0%, *P*=0.79; the Middle East: *I*^2^ = 74%, *P*=0.02). The random-effects model was used. The fasting blood glucose in the curcumin group was lower than that in the control group Asia subgroup (SMD: −0.57, 95% CI: −0.79 to −0.36, *P* < 0.00001). However, the difference between the curcumin group and the control group in the Middle East subgroup was of no statistical significance (SMD: 0.04, 95% CI: −0.50 to 0.58, *P*=0.89). The summary results also showed that the difference between two groups was of no statistical significance (SMD: −0.28, 95% CI: −0.62 to 0.06, *P*=0.11) (Figure 7).

Two RCTs [11, 16–19] reported fasting insulin. Due to the high heterogeneity (*I*^2^ = 96%, *P* < 0.00001), the statistical analysis was abandoned according to the Cochrane Handbook for Systematic Reviews of Interventions. Panahi et al. [16–19] found no significant difference in fasting insulin levels between the two groups (*P* > 0.05), while Chuengsamarn et al. [11] found that the curcumin group had lower fasting insulin levels (*P* < 0.05).

#### 3.5.3. Blood Lipid

**Figure 8:**
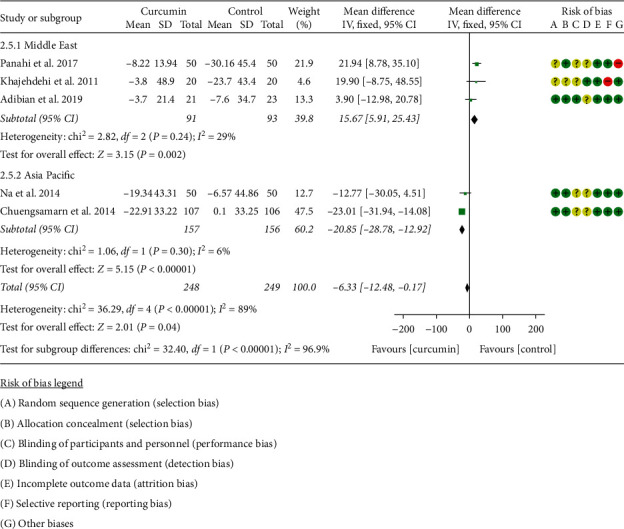
LDL-C.

Five RCTs [11, 16–22, 34] including 248 participants in the curcumin group and 249 participants in the control group reported LDL-C and HDL-C. Due to the high heterogeneity, 5RCTs were subdivided into two subgroups according to the region of the patients. After subdivision, the heterogeneity was low in each subgroup (Asia: *I*^2^ = 6%, *P*=0.30; the Middle East: *I*^2^ = 29%, *P*=0.24) among the RCTs. The fixed-effects model was used. The LDL-C in the curcumin group was lower than that in the control group in the Asia subgroup (WMD: −20.85, 95% CI:−28.78 to −12.92, *P* < 0.00001), but it was higher in the Middle East subgroup (WMD: 15.67, 95% CI: 5.91 to 25.43, *P*=0.002) (Figure 8).

**Figure 9:**
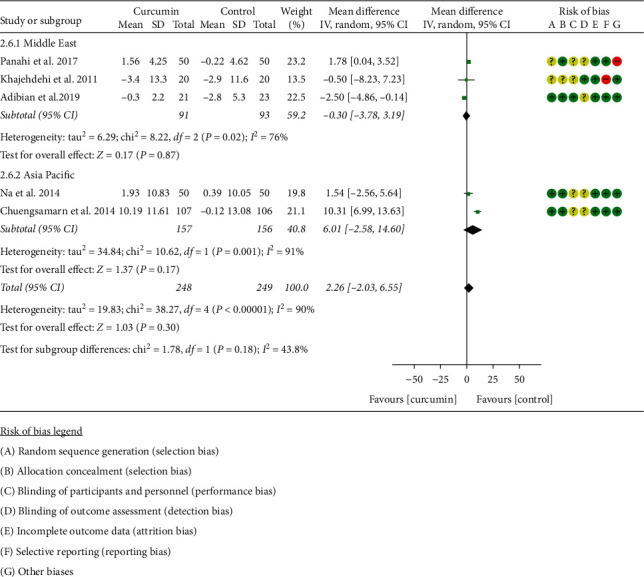
HDL-C.

The RCTs in HDL-C were also subdivided into two subgroups according to the region of the patients for the same sake. After subdivision, the heterogeneity was still high in the two subgroups (Asia Pacific: *I*^2^ = 91%, *P*=0.001; the Middle East: *I*^2^ = 76%, *P*=0.02) among the RCTs. The random-effects model was used. The difference in HDL-C between two groups was of no statistical significance in each subgroup (the Middle East: WMD: −0.30, 95% CI: −3.78 to 3.19, *P*=0.87; Asia: WMD: 6.01, 95% CI: −2.58 to 14.60, *P*=0.17). The summary result also showed that the difference between two groups was of no statistical significance (WMD: 2.26, 95% CI: −2.03 to 6.55, *P*=0.30) (Figure 9).

#### 3.5.4. Adiponectin

**Figure 10:**
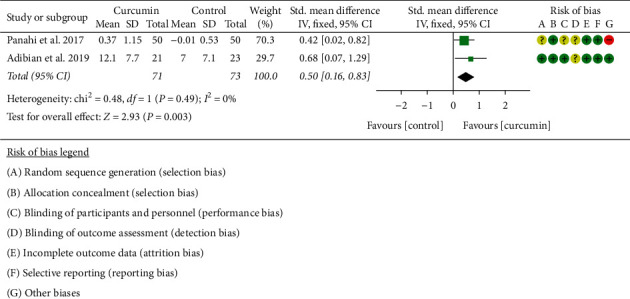
Adiponectin.

Three RCTs [11, 16–19, 34] reported adiponectin. The data of Chuengsamarn et al. [11] could not be extracted, so only the data of 2 RCTs were extracted for analysis. The heterogeneity between RCTs is low (*I*^2^ = 0%, *P*=0.49), so a fixed-effects model is used. Compared with the control group, the curcumin group had higher adiponectin (SMD: 0.50, 95% CI: 0.16 to 0.83, *P*=0.003) (Figure 10).

### 3.6. Adverse Events

Four RCTs [11, 16–19, 22, 31, 32] reported adverse events. Two RCTs [16–19, 22] showed that there are no severe related side effects in two groups. One RCT [11] showed that four patients in the curcumin group and four in the control group exhibited adverse events. Asadi et al. [31, 32] reported 2 cases of stomach pain in the first few days, but did not specify the group.

### 3.7. Sensitivity Analysis Results

**Figure 11:**
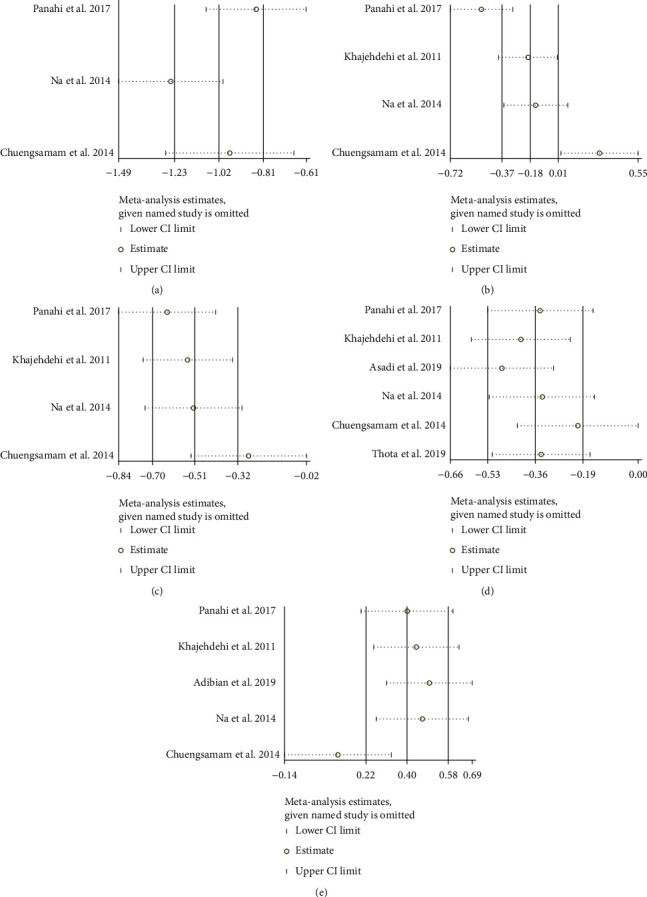
Sensitivity analysis results: (a) HOMA-IR; (b) TC; (c) TG; (d) FBG; (e) HDL-C.

Sensitivity analysis was performed for 5 outcomes: HOMA-IR, TC, TG, FBG, and HDL-C. (1) In the outcome “HOMA-IR,” after we omitted Na et al. [20, 21], we found that the estimate of the result moved out of the lower limit of 95% CI (Figure 11(a)). (2) In the outcome “TC,” after we omitted Panahi et al. [16–19], we found that the estimate of the result moved out of the lower limit of 95% CI, while after omitting Chuengsamarn et al. [11], the estimate of the result moved out of the upper limit of 95% CI (Figure 11(b)). (3) In the outcome “TG,” after omitting Chuengsamarn et al. [11], the estimate of the result moved out of the upper limit of 95% CI (Figure 11(c)). (4) In the outcome “FBG,” no matter which study was removed, the results were not significantly changed, suggesting that the heterogeneity may not come from RCT (Figure 11(d)). (5) In the outcome “HDL-C,” after omitting Chuengsamarn et al. [11], the estimate of the result moved out of the lower limit of 95% CI (Figure 11(e)). The abovementioned RCTs may be the source of heterogeneity of corresponding outcomes.

### 3.8. Publication Bias Detection

**Figure 12:**
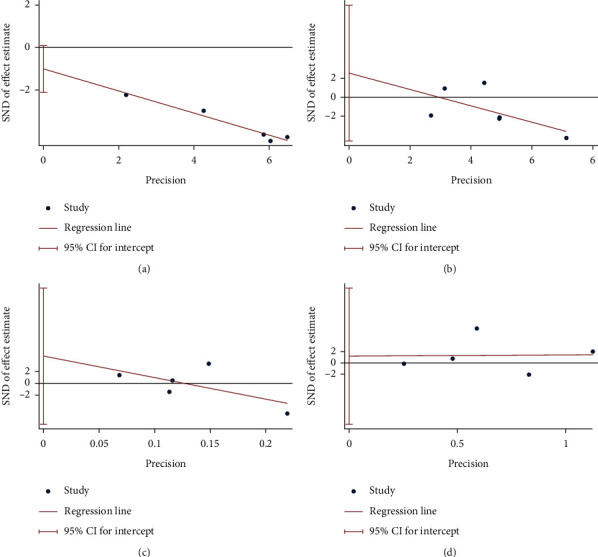
Publication bias detection (a) HbA1c; (b) FBG; (c) LDL-C; (d) HDL-C.

(1) HbA1c:the results of HbA1c indicate that there may be publication bias (*P*=0.061) (Figure 12(a)). (2) FBG: the publication bias detection suggests that there may be no publication bias (*P*=0.381) (Figure 12(b)). (3) LDL-C: the publication bias detection suggests that there may be no publication bias (*P*=0.296) (Figure 12(c)). (4) HDL-C: the publication bias detection suggests that there may be no publication bias (*P*=0.776) (Figure 12(d)).

## 4. Discussion

### 4.1. Main Findings

The HOMA-IR of the curcumin group is lower in Asia and the Middle East subgroups. The HbAlc in the curcumin group is lower than the control group. The TC, TG, and fasting blood glucose level of the curcumin group is lower in the Asia subgroup, while in the Middle East, the difference was of no statistical significance. For BMI, fasting insulin, and HDL-C level, there is no strong evidence that which one is better. Interestingly, although the LDL-C level of the curcumin group in the Asia subgroup is lower than that of the control group, the LDL-C level of the curcumin group was higher than the control group in the Middle east subgroup.

### 4.2. Overall Completeness and Applicability of Evidence

Most of RCTs come from Asia Pacific (especially Southeast Asia, like China and Thailand) and the Middle East (mainly Iran). Due to the lack of RCTs from all over the world, the applicability of the findings is limited.

### 4.3. Discussion of the Source of Heterogeneity

Most outcomes have heterogeneity, so this study conducted a sensitivity analysis to find the source of heterogeneity. Sensitivity analyses were performed for 5 outcomes: HOMA-IR, TC, TG, FBG, and HDL-C.

For outcome “HOMA-IR,” compared with the other two RCTs, the dose of curcuminoids in Na et al. [20, 21] was lower (300 mg), while in Panahi et al. [16–19] it was 1000 mg, and in Chuengsamarn et al. [11] it was 1500 mg. Therefore, the heterogeneity of HOMA-IR may be related to the drug dose. For the outcome “TC,” the mean age in Panahi et al. [16–19] is lower, which may be the source of heterogeneity. For the outcome “TC,” “TG,” and “HDL-C,” Chuengsamarn et al. [11] prepared curcumin monomer, while the preparations of several other RCTs are curcuminoids or turmeric, which suggests that different drug preparation methods may be the source of heterogeneity. For the outcome “FBG,” no matter which study was removed, the results were not significantly changed, suggesting that the heterogeneity may not come from RCT.

In addition to the above possible sources of heterogeneity, heterogeneity may also come from ethnic differences, regional differences, gender differences, body size differences, and so on. More relevant RCTs are needed to be conducted in more diverse subgroups to reduce the heterogeneity of the research and stabilize the conclusions.

### 4.4. Novelty of This Research

This systematic review and meta-analysis showed that curcumin can improve HOMA-IR and HbAlc, especially in Asian patients and the Middle Eastern patients. In terms of blood lipids, curcumin can reduce TC, TG, and fasting blood glucose level in Asian patients, but does not improve in the Middle East patients significantly. Curcumin may lower the LDL-C level of Asian patients. Interestingly, after curcumin intervenes in the Middle Eastern patients, their LDL-C level has increased. This is something that needs attention in the future. Demmers et al. [35] estimated the available scientific data on the effectiveness and safety of medicinal food plants in the treatment of impaired glucose tolerance. It included an RCT of curcumin extract intervention for diabetes and found that the fasting blood glucose at 2 hours after intervention showed statistical significance after 3, 6, and 9 months (*P* < 0.01). In addition, the curcumin extract intervention (HbA1c) value showed statistical significance after 3, 6, and 9 months (*P* < 0.01). HOMA-IR after curcumin extract intervention showed statistical significance after 6 months and 9 months (*P* < 0.05 and *P* < 0.01). It shows that curcumin has shown a reliable result that is effective in treating impaired glucose tolerance. Compared with Demmers et al.'s [35] research, our study is mainly devoted to exploring the interventional effects of curcumin and turmeric extracts on T2DM. We included more curcumin in the treatment of T2DM-related RCTs, and the evidence for improving the outcome of multiple related indicators of glucose tolerance is more sufficient. Compared to Marton et al.'s [36] review, (1) in this study, the RCT data (HOMA-IR, HbAlc, and so on) of curcumin treatment of T2DM were statistically analyzed (meta-analysis). (2) This study also conducted a subgroup analysis based on regions. (3) We adopted stricter screening criteria and merged the records belonging to the same RCT. (4) Sensitivity analysis was conducted in this study, which can more accurately locate the main source of heterogeneity. (5) The publication bias assessment was carried out in this study, and the publication bias of outcomes was excluded. In terms of improving metabolism, Roshanravan et al. [37] found the protective effect of supplementing *Crocus sativus* L. on hyperlipidemia and hyperglycemia through systematic reviews and meta-analysis studies. Our research also found that curcumin has the same clinical effect in improving the metabolism of diabetes.

### 4.5. The Strengths of This Review

This registered systematic review and meta-analysis is the first one that comprehensively evaluated the previous RCT of curcumin on T2DM and underwent a subgroup analysis to assess the applicable population. It also collected the detailed data from each RCT and a comprehensive assessment of risk of bias was conducted.

### 4.6. The Limitations of This Review

RCTs from Asia and the Middle East account for a large proportion, which affects the applicability of the findings. The quantity and quality of RCTs are also not high in this review; several subgroups only include one RCT. Only 453 participants were included in this review, which may also impact the applicability of the findings. Meanwhile, half of RCTs [16–19, 22] have an unclear risk of bias in randomization, and one of the RCTs [22] has a high risk of bias in selective reporting; this may also influence the interpretation of the results. In addition, most of the outcomes have a high heterogeneity (such as HOMA-IR, TC, and TG) that cannot be eliminated by appropriate subgroup analysis. This also affected the applicability of the findings. The heterogeneity may come from the potential discrepancies in the pharmacological effects of various curcumin preparations, which may result from different standardizations of curcumin manufacturing process, dosage, duration of treatment, units of laboratory tests, and races of the selected patients or other places.

### 4.7. Implications for Future

This systematic review and meta-analysis found that curcumin may improve the HOMA-IR and fasting blood glucose of individuals in Asia and the Middle East. Curcumin may also improve the glycemic control in patients with T2DM. For blood lipids, curcumin may decrease the TC and TG in Asian T2DM patients, while its effects on the Middle Eastern T2DM patients may not be as significant as that on Asians; interestingly, according to the results, curcumin may decrease the LDL-C level in Asian T2DM patients, while it has the opposite effect on Middle Eastern patients. This may be related to ethnic differences, and more research is needed to amend or confirm it. In addition, in the aspect of decreasing BMI and fasting insulin, and improving HDL-C, curcumin may not have an advantage over the control group. However, due to the lack of evidence, more RCTs are needed. Last but not the least, for safety, there are no serious adverse events reported in RCTs, and the occurrence of adverse events in curcumin groups is the same as that of the placebo control group; therefore, it can be considered as a safety treatment based on current evidence.

In summary, curcumin may be more effective in Asia. In addition, for clinical practices, curcumin may be recommended as an adjunct to the treatment of T2DM patients to improve insulin resistance and glycemic control and reduce blood lipids. For Middle Eastern T2DM patients, the appropriate dosage, usage, and so on are yet to be confirmed. For future research, more RCTs about the adverse events and the T2DM-related outcomes are needed to revise or validate the findings in this review. The RCTs containing the data of the patients of other countries or regions around the world are also needed to expand the applicability of the results [38].

## 5. Conclusion

Based on the current evidence, curcumin may assist in improving insulin resistance, glycemic control, and decrease in TG and TC in patients with T2DM.

## Figures and Tables

**Table 1:** Search strategy for PubMed.

Database	Search strategy
PubMed	(Curcumin OR Turmeric Yellow OR Yellow, Turmeric OR Diferuloylmethane) AND (Type 2 diabetes mellitus OR Diabetes Mellitus, Noninsulin-Dependent OR Diabetes Mellitus, Ketosis-Resistant OR Diabetes Mellitus, Ketosis Resistant OR Ketosis-Resistant Diabetes Mellitus OR Diabetes Mellitus, Non Insulin Dependent OR Diabetes Mellitus, Non-Insulin-Dependent OR Non-Insulin-Dependent Diabetes Mellitus OR Diabetes Mellitus, Stable OR Stable Diabetes Mellitus OR Diabetes Mellitus, Type II OR NIDDM OR Diabetes Mellitus, Noninsulin Dependent OR Diabetes Mellitus, Maturity-Onset OR Diabetes Mellitus, Maturity Onset OR Maturity-Onset Diabetes Mellitus OR Maturity Onset Diabetes Mellitus OR MODY OR Diabetes Mellitus, Slow-Onset OR Diabetes Mellitus, Slow Onset OR Slow-Onset Diabetes Mellitus OR Type 2 Diabetes Mellitus OR Noninsulin-Dependent Diabetes Mellitus OR Noninsulin Dependent Diabetes Mellitus OR Maturity-Onset Diabetes OR Diabetes, Maturity-Onset OR Maturity Onset Diabetes OR Type 2 Diabetes OR Diabetes, Type 2 OR Diabetes Mellitus, Adult-Onset OR Adult-Onset Diabetes Mellitus OR Diabetes Mellitus, Adult Onset) AND (randomized controlled trial [pt] OR controlled clinical trial [pt] OR placebo [tiab] OR drug therapy [sh] OR trial [tiab] OR groups [tiab] OR clinical trials as topic [mesh: noexp] OR Clinical Trial OR random∗ [tiab] OR random allocation [mh] OR single-blind method [mh] OR double-blind method [mh] OR cross-over studies) NOT (animals [mh] NOT humans [mh])

**Table 2:** The characteristics of the included studies.

Study	Register ID	Country	Sample size		Intervention		Relevant outcomes	Duration
			Trial group	Control group	Trial group	Control group		

Panahi et al. [16–19]	IRCT201505301165N4	Iran	50	50	Curcuminoids (C3 Complex®) 1000 mg + 10 mg piperine	Placebo + 10 mg piperine	BMI, TG, TC, LDL-C, HDL-C, HOMA-IR, HbAlc, fasting blood glucose, fasting insulin, adiponectin adverse events	12 weeks

Na et al. [20, 21]	ISRCTN85826075	China	50	50	Curcuminoids 300 mg with no changes to patients' previous drug medication	Placebo with no changes to patients' previous drug medication	TG, TC, LDL-C, HDL-C, HOMA-IR, HbAlc, fasting blood glucose	12 weeks

Chuengsamarn et al. [11]	NCT01052597	Thailand	107	106	Curcumin 1500 mg with no oral antidiabetes or insulin injection	Placebo (starch) 1500 mg with no oral antidiabetes or insulin injection	BMI, TG, TC, LDL-C, HDL-C, HOMA-IR, HbAlc, fasting blood glucose, fasting insulin, adiponectin, adverse events	24 weeks

Khajehdehi et al. [22]	—	Iran	20	20	Turmeric 1500 mg with no changes to patients' previous drug medication	Placebo with no changes to patients' previous drug medication	TG, TC, LDL-C, HDL-C, HOMA-IR, HbAlc, fasting blood glucose, adverse events	8 weeks

Asadi et al. [31, 32]	IRCT20140413017254N5	Iran	40	40	Nanocurcumin 80 mg with no changes to patients' previous hypoglycemic drugs	Placebo with no changes to patients' previous hypoglycemic drugs	BMI, HbAlc, fasting blood glucose, adverse events	8 weeks

Thota et al. [33]	ACTRN12615000559516	Australia	15	16	Curcumin 1000 mg	Placebo (2000 mg corn oil)	HbAlc, TC, LDL-C, HDL-C, fasting blood glucose, adverse events	12 weeks

Adibian et al. [34]	NCT02529969	Iran	21	23	Curcumin 1500 mg with no changes to patients' previous drug medication	Placebo (rice flour) 1332 mg with no changes to patients' previous drug medication	BMI, TG, TC, LDL-C, HDL-C, fasting blood glucose, fasting insulin, adiponectin	10 weeks

Study	BMI		Mean age (years)		Inclusion criteria	Exclusion criteria
	Trial group	Control group	Trial group	Control group		
Panahi et al. [16–19]	26.53 ± 2.32	27.33 ± 1.58	43 ± 8	41 ± 7	Diagnosis of T2DM based on fasting plasma glucose (FPG) ≥ 126 mg/dL, glycated hemoglobin (HbA1C) ≥ 6.5%, or the use of antidiabetic treatments	Pregnancy or breastfeeding, lack of possibility to give an informed consent, participation in a concomitant trial, presence of malignancies, chronic liver disease (alanine aminotransferase levels three times the upper limit of normal value range), renal failure (serum creatinine ≥ 2.0 mg/dL or being on dialysis), chronic inflammatory diseases such as rheumatoid arthritis and acute infections, endocrine diseases other than T2DM (e.g., hypothyroidism or hyperthyroidism), obsessive compulsive disorder, hyperglycemia due to secondary causes, receiving hormone therapy or other herbal medicines, hypersensitivity to the study medication, and lack of compliance with the study medication

Na et al. [20, 21]	27.12 ± 2.26	27.42 ± 3.04	55.42 ± 6.40	54.72 ± 8.34	(1) Aged 18–65 years, both male and female (2) Type 2 diabetes with fasting blood glucose greater than or equal to 7.0 mmol/L or postprandial blood glucose greater than or equal to 11.1 mmol/L (3) Current optimal therapeutic regimens lasting for at least 6 months	(1) A history of type 1 diabetes, malignancies, thyroid, or any other endocrine diseases likely to interfere with the study (2) Diabetic ketosis acidosis and infection in recent 3 months (3) Pregnancy or breastfeeding (4) Incomplete information or unwillingness to attempt to comply with the intervention

Chuengsamarn et al. [11]	27.09 ± 0.52	26.84 ± 0.42	59.16 ± 11.04	59.58 ± 10.71	(1) Diabetic patients aged 35 years or older and did not use insulin during the first 5 years of treatment after being diagnosed (with or without symptoms listed in the following inclusion criteria) (2) Patients with hyperlipidemia (cholesterol ≥ 200 mg/dl, TG ≥ 150 mg/dl, LDL ≥ 100 mg/dl, and HDL ≥ 35 mg/dl) (3) Patients with hypertension (blood pressure ≥ 130/85 mmHg or take hypertensive drugs) (4) Obesity (BMI ≥ 25)	(1) Current diagnosis of secondary peripheral arterial disease (PAD) (except listed in the inclusion criteria item 1–4) (2) Current diagnosis of cardiovascular disease, i.e., coronary arterial disease and cerebrovascular disease (3) Current diagnosis of end-stage renal function with serum creatinine >2.0 mg/dl or on renal dialysis (4) Current diagnosis of cirrhosis with ALT ≥3 times the normal range

Khajehdehi et al. [22]	—	—	52.9 ± 9.2	52.6 ± 9.7	Patients with overt type 2 diabetic nephropathy (proteinuria ≥500 mg/day), who had normal kidney function and well-controlled blood pressure by ACE-I and/or angiotensin receptor blockers	Patient with recurrent or relapsing urinary tract infection, bacteriuria, pyuria and/or haematuria, or who failed to sign a written informed consent when risks associated with the trial was carefully outlined for them
Asadi et al. [31, 32]	31.1 ± 4.2	30.8 ± 3.8	53.3 ± 6.5	54.6 ± 6.2	Age between 30 and 60 years, desire to participate in the study, body mass index between 25 and 39.9, noninsulin type 2 diabetic patients, detection of mild sensorimotor polyneuropathy by using the Toronto questionnaire (score 6–8)	Follow a special diet during last month, sensitivity to curcumin, pregnancy and lactation, took any nutritional supplement, vitamin and mineral supplement last month, history of gastrointestinal ulcer and bile duct, neuropathies other than the sensory-motor polyneuropathy diabetic diagnosed by a neurologist, taking gabapentin and any medication, diseases such as cancer, liver, kidney, autoimmune diseases, inflammatory, thyroid and nervous and cardiovascular diseases are diagnosed, and drug use associated with these diseases


Thota et al. [33]	30.9 ± 1.2	31.9 ± 1.7	55 ± 2.8	50 ± 2.5	(1) Age: 30–70; gender: both male and female (2) No participation in any clinical trial for at least 3 months (3) An HbA1c of 5.7%–6.4% (4) Impaired glucose tolerance (IGT) (5) 2-hour OGTT plasma glucose greater than or equal to 7.8 mmol/land <11.1 mmol/L (6) Impaired fasting glucose (IFG) (7) Fasting plasma venous glucose measurement 6.1–6.9 mmol/L (8) 12 or more score or high-risk individuals in AUSDRISK assessment tool (9) BMI between 25 and 45	(1) Pregnancy or lactation (2) Established type 2 diabetes (3) Allergic to sea foods (4) People with gall bladder problems (5) People currently on medication with erythropoietin (6) People with anaemia (7) People with pace maker implants (8) Currently on medication with aspirin and warfarin (9) History of severe neurological diseases or seizures (10) History of new investigational drug three months prior to this trial (11) Consuming more than 2 servings of oily fish per week (12) Taking regular dietary supplements known to influence blood glucose level (13) People taking regular vitamin C supplements (14) Unwilling to fast for 10 hr before obtaining blood sample. People currently on medication with clopidogrel, ibuprofen, naproxen, dalteparin, enoxaparin, and heparin

Adibian et al. [34]	—	—	Around 58	60 ± 7	(1) Tendency to participate (2) Age range of 40–65 (3) Suffering from diabetes type 2 (for 1 to 10 years) (4) BMI 18/5–30 (5) Patients with diabetes who administer oral hypoglycemic agents and who do not use them	(1) Patients with liver diseases (2) Patients with kidney diseases (3) Patients with inflammatory diseases (4) Patients with liver diseases (5) Administering herbal agents (6) Administering multivitamins and minerals in past 3 months

## Data Availability

All data generated or analyzed during this study are included in this published article.

## Supplementary Materials

Supplementary Materials “CRD42018089528” is the protocol of our systematic review and meta-analysis. The “PRISMA 2009 checklist” is a standardized checklist that make sure that systematic reviews and meta-analyses are implemented according to this standard.
